# Plant response to environmental conditions: assessing potential production, water demand, and negative effects of water deficit

**DOI:** 10.3389/fphys.2013.00017

**Published:** 2013-02-18

**Authors:** François Tardieu

**Affiliations:** Laboratoire d'Ecophysiologie des Plantes sous Stress Environnementaux, Institut National de la Recherche AgronomiqueMontpellier, France

**Keywords:** stress, drought, temperature, intercepted light, plant performance

## Abstract

This paper reviews methods for analyzing plant performance and its genetic variability under a range of environmental conditions. Biomass accumulation is linked every day to available light in the photosynthetically active radiation (PAR) domain, multiplied by the proportion of light intercepted by plants and by the radiation use efficiency. Total biomass is cumulated over the duration of the considered phase (e.g., plant cycle or vegetative phase). These durations are essentially constant for a given genotype provided that time is corrected for temperature (thermal time). Several ways of expressing thermal time are reviewed. Two alternative equations are presented, based either on the effect of transpiration, or on yield components. Their comparative interests and drawbacks are discussed. The genetic variability of each term of considered equations affects yield under water deficit, via mechanisms at different scales of plant organization and time. The effect of any physiological mechanism on yield of stressed plants acts via one of these terms, although the link is not always straightforward. Finally, I propose practical ways to compare the productivity of genotypes in field environments, and a “minimum dataset” of environmental data and traits that should be recorded for that.

## Introduction

Plants transform light and CO_2_ into biomass. This occurs during a given period of time, the duration of which depends essentially on air temperature and on the earliness of the considered genotype. During the same period of time, the plant requires an amount of water that depends on environmental conditions (light, air humidity, and wind) and on plant traits such as stomatal conductance and leaf area. It follows that the biomass accumulated by a plant primarily depends on environmental conditions, but also depends on plants traits with their genetic variability. The objective of this paper is to provide a basis for analyzing yield from environmental conditions, thereby enabling characterization of the differences in behavior between genotypes. This basis is the common ground of most existing crop models (Sinclair et al., 1976; Brisson et al., 2003; Hammer et al., 2010), and of global analyses of the effects of climate change on plant performance (Brisson et al., 2010; Lobell et al., 2011).

## Variability of yield depending on light and water availability

### The maximum yield that can be obtained in a given field depends on the amount of intercepted light

Biomass accumulation is proportional to the amount of light in the photosynthetically active radiation (PAR) domain that the plant intercepts over a period of time (Monteith, 1977). Why is biomass accumulation proportional to light while photosynthesis is not? Photosynthesis depends on light intensity, with a relationship that is approximately linear for low light intensities (about 0–700 μmol m^−2^ s^−1^), but curvilinear at higher intensities (Farquhar et al., 1980). The linear relationship between biomass and light is due first to lowest leaves of the canopy being shaded, so they receive light in the range where photosynthesis is nearly proportional to light. Second, the light intensity only exceeds 700 μmol m^−2^ s^−1^ during the late morning and early afternoon, and is below this value during the rest of the day. Hence, the resulting relationship between photosynthesis and light is linear at the field level and during the entire day.

**Figure 1:**
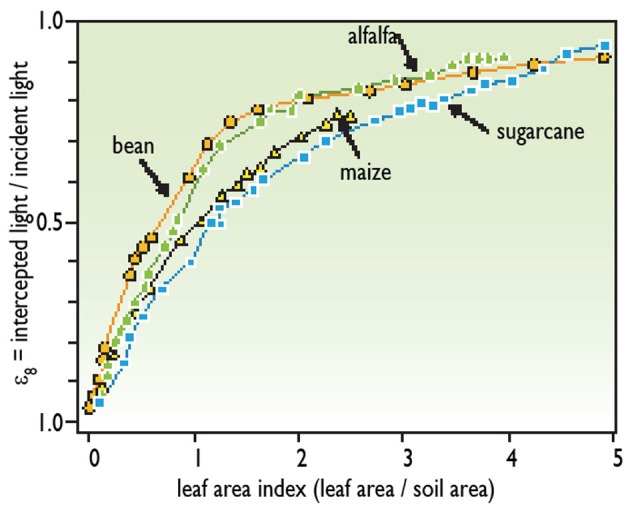
**Relationship between leaf area and light interception (redrawn from Gosse et al., 1986)**.

The biomass accumulation by a crop on a given day (Bio_*i*_) depends on:

- The amount of light on the considered day in the range of wavelengths used by photosynthesis [photosynthetic photon flux density (PPFD_*i*_)]. Most light sensors directly record the amount of light in this range.
- The proportion of light that is intercepted by plant leaves. The light that reaches the soil is not used for photosynthesis. The proportion of intercepted light depends on leaf area on the considered day and is characterized by the leaf area index (LAI), which is the number of layers of leaves per unit soil area. For instance, an LAI of 1 corresponds to a plant canopy with 1 m^2^ of leaves per m^2^ of soil. The proportion of light intercepted by plants increases with LAI (Figure 1) until an LAI of 4 or 5 depending on the species. At a higher LAI, nearly all the available light is intercepted (there are no spots of unused light on the soil). The relationship between LAI and the proportion of intercepted light differs among species (Figure 1). Plants intercept more light per unit leaf area in species with sub-horizontal leaves, such as clover or cassava, than in species with erect leaves, such as cereals.
- The efficiency of transformation of intercepted light into biomass, which depends essentially on the photosynthesis rate of leaves (Gosse et al., 1986; Brisson et al., 2003; Hammer et al., 2010). This efficiency differs between species; it is maximal in C_4_ species such as maize, sorghum, or millet, which have a very efficient photosynthetic apparatus. It is roughly similar in all C_3_ species that are neither legumes nor oil-rich seeds, such as wheat or rice. It is lower in species that have a special metabolism such as legumes, which use part of the photosynthesized sugars for nitrogen fixation (Gosse et al., 1986). It is also lower in species with oil-producing seeds, which have high energy content per unit biomass of seeds.

These three effects can be summarized in a simple equation:

(1) $${\text{Bio}}_{i}={\text{PPFD}}_{i}\cdot \varepsilon_{a}\cdot \varepsilon_{b}$$

where Bio_*i*_ is the biomass accumulated on a given day, PPFD is the photosynthetic photon flux density, also called light intensity (in W m^−2^ or μmol m^−2^ s^−1^), ε_*a*_ is the proportion of intercepted light (in percentage), and ε_*b*_ is the efficiency of transformation (in g per unit light intensity). The biomass accumulated during the whole season (Bio_tot_) is the sum of biomasses accumulated each day:

(2) $${\text{Bio}}_{\text{tot}}=\sum \left({\text{PPFD}}_{i}\cdot \varepsilon_{a}\cdot \varepsilon_{b}\right)$$

The yield is the fraction of Bio_tot_ that is transferred to harvested organs, such a grains or tubers. The proportion of harvested biomass divided by the total biomass is usually termed the “harvest index” (HI), expressed as a percentage:

(3) $$\text{Yield}=\sum \left({\text{PPFD}}_{i}\varepsilon_{a}\cdot \varepsilon_{b}\right)\text{HI}$$

This expression (Monteith, 1977) is very useful for analyzing the yield performance of a given genotype and to compare genotypes. In particular:

- The best genotypes are those that give a high priority to the harvested organs in the biomass partitioning. A large part of the genetic progress of several species has consisted of increasing HI (Lopez Pereira et al., 1999; Duvick, 2005).
- In contrast, the efficiency of conversion of intercepted light into biomass is less variable between genotypes of a given species. In particular, the genetic progress of photosynthesis has been slow, if not negligible (Bolanos and Edmeades, 1993a; Lopez Pereira et al., 1999; Lee and Tollenaar, 2007). Some genetic programs have nevertheless increased RUE (Rebetzke et al., 2002).
- Another large source of genetic variation is the proportion of light that is intercepted by plants. The genetic variation in leaf area then translates into a change in accumulated biomass and, in turn, into yield in the range of LAI from 1 to 5 (Gosse et al., 1986; Hammer et al., 2010). However, confounding effects, as analyzed below, may obscure this relationship.
- Finally, yield largely depends on the number of days during which biomass accumulates (term Σ in Equation 3). It is intuitive that the longer the crop cycle the higher the maximum potential yield. This number of days depends on the temperature sensed by plants during the crop cycle. Increasing temperature tends to cause shorter crop cycles, thereby decreasing the potential yield. It also depends on the genotype via two traits: first the earliness of the considered genotype, which defines the time for flowering and the duration of the period between flowering and maturity; and second the degree of maintenance of this period under stressing conditions.

### An alternative way of expressing yield as a function of water availability and water-use efficiency

Water availability does not appear directly in the analysis presented above, because water is not involved *per se* in the process of biomass accumulation. In contrast to light which has a nearly proportional effect on the accumulation of biomass, water “only” serves to allow biomass accumulation to occur in good conditions by favoring stomatal opening, organ growth, and plant metabolism. In order to express yield as a function of water-use, an alternative expression of yield has been proposed by Passioura (1977). This states that the biomass accumulation on 1 day depends on the transpiration rate multiplied by the water-use efficiency (WUE) i.e., the ratio of biomass accumulation to transpiration. As in Equation (2), the biomass accumulated over the plant cycle is the sum of that accumulated every day of the cycle. The yield is the fraction of the accumulated biomass that is transferred to harvested organs, such a grains or tubers (i.e., the HI):

(4) $$\text{Yield}=\sum \left(T_{i}\cdot {\text{WUE}}_{i}\right)\text{HI}$$

where *T*_*i*_ is the transpiration on day *i*, WUE_*i*_ is the WUE on day *i* and Σ indicates that the biomass is accumulated over the whole crop cycle.

#### Transpiration rate

*T*_*i*_ changes every day depending on evaporative demand and on leaf area. Evaporative demand depends essentially on light, on the degree of water saturation of the air, measured as vapor pressure deficit (VPD), and on wind speed (Sinclair et al., 1976; Brisson et al., 2003). Most weather stations provide the evaporative demand, termed “potential evapotranspiration” or “reference evapotranspiration.” Otherwise, evaporative demand can be calculated using a spreadsheet by using Penman's formula.

Leaf area affects the transpiration rate in the same way as it affects the intercepted light (Figure 1). Thus, transpiration is nearly proportional to leaf area for low LAI, and saturates for LAI higher than 3 or 4.

(5) $$T=ET_{r}\cdot \varepsilon_{a}$$

where *ET*_*r*_ is the reference evapotranspiration, as provided by a weather station or calculated using Penman's formula, and ε_*a*_ is the proportion of transpiration of the studied field to the reference evapotranspiration, which has the same value as that in Equation (1).

The root system also affects the transpiration rate, via several traits such as total root length, rooting depth, or the hydraulic conductivity of roots. It should be noted that this is the case only if roots have access to a large volume of soil. In contrast, an increase in root length has virtually no effect in a shallow soil. Two breeding programs for drought have resulted in the surprising result that the root length was reduced in drought-tolerant genotypes compared with drought-sensitive ones (Bolanos et al., 1993; Bruce et al., 2002).

#### Water-use efficiency

WUE is defined here as the ratio of the biomass accumulated on 1 day to the transpiration rate on the same day. Defined in this way, it is difficult and tedious to measure. A surrogate measurement consists of the ratio between the photosynthetic rate and the transpiration rate, or between photosynthesis and stomatal conductance as measured using gas exchange equipment. The latter can be measured indirectly via the ratio of two natural isotopes of carbon in leaves or grains (carbon isotope discrimination, often called D^13^C), providing rapid estimates with a high throughput.

**Figure 2:**
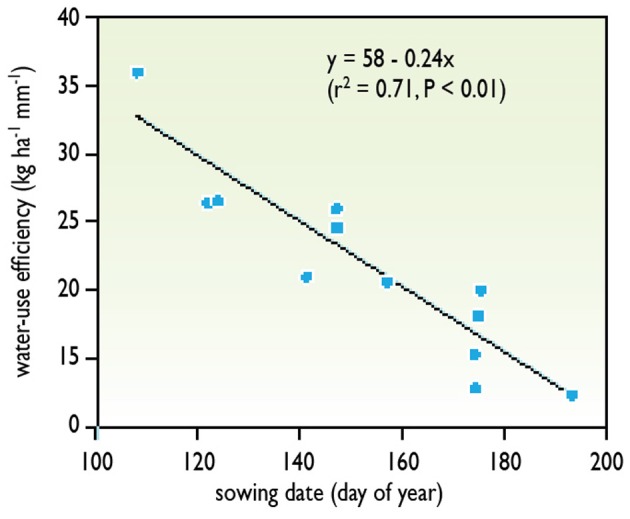
**An example of variation of WUE during the crop cycle.** The later in the growing period, the higher is the evaporative demand. Because the accumulated biomass does not increase in the same proportion, the WUE is lower during periods with high evaporative demand. Data obtained with wheat lines sown at different times of the year in Australia (redrawn from Richards et al., 2002).

Environmental conditions greatly affect WUE (defined as in Equation 6). In particular, WUE decreases when evaporative demand increases, because transpiration is higher at high evaporative demands for a given photosynthesis. It follows that WUE is higher in regions with wet air, and that crops that are grown during winter or during rainy seasons have a higher WUE than those grown during summer or during dry seasons (Figure 2). Large differences in WUE exist between species. WUE is higher in C_4_ species, such as maize, sorghum, or millet, than in C_3_ species. It is noteworthy that the method based on carbon isotope discrimination cannot be used in C_4_ species.

Finally, it should be noted that the concept of WUE, and therefore Equation (4), can be misleading, depending on the definition that is taken for WUE and on the time scale (Blum, 2005, 2009). Depending on the study or paper, WUE is defined in different ways that are not equivalent, have different genetic variabilities, and respond differently to environmental conditions. Defined as the ratio of photosynthesis to transpiration, WUE has a lower genotype-by-environment interaction (GEI), but it cannot be used directly as the efficiency of transforming transpiration into biomass. At the other extreme, WUE can be defined at the scale of the crop cycle as the ratio of the total biomass (or yield) to the total transpiration. It should be noted that, in this case, WUE is not a direct consequence of stomatal and photosynthetic functioning, and is affected by growth conditions. For instance, a short stress that causes abortion of reproductive organs affects total biomass accumulation, with a lesser effect on transpiration. Therefore, it affects WUE at the whole cycle scale, although it has a small effect on gas exchange.

### A third expression of yield as a series of yield components: roles of individual phases of the crop cycle

Agronomists have long expressed yield by a multiplicative series of yield components: the number of plants per m^2^; the number of immature reproductive organs per plant (e.g., the number of seeds per tiller multiplied by the number of tillers per plant, or the number of tubers per stem multiplied by the number of stems per plant); the proportion of non-aborted reproductive organs; and the individual weight of seeds or tubers. Thus:

(6) $$\text{Yield}=N(1-\text{A})W_{r}$$

where *N* is the number of immature reproductive organs (e.g., ovules) per unit area, A is the proportion of aborted reproductive organs, and *W*_*r*_ is the mean weight of individual reproductive organs.

**Figure 3:**
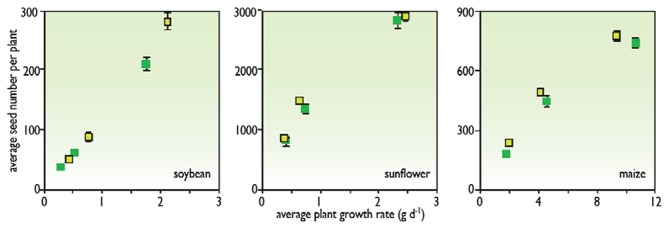
**The relationship between seed number per plant at maturity and average plant growth rate at flowering time in soybeans, sunflowers, and maize (redrawn from Vega et al., 2001)**.

This expression has the advantage of breaking down the yield into several phase of the crop cycle. The setting of reproductive organs occurs during the pre-flowering time, the proportion of non-aborted reproductive organs is determined during a phase around flowering, and the individual weight of seeds or tubers is determined between flowering and maturity. Therefore, it is possible to express the result of each phase as a function of environmental conditions during that phase. For instance, one can relate the abortion rate to the water availability during the same period (Claassen and Shaw, 1970) or to the biomass accumulation during that period (Vega et al., 2001) (Figure 3). In the same way, seed number usually correlates well to the intercepted light during the pre-flowering period. These relationships help to identify the behaviors of genotypes, which can either have common behaviors (common relationships) or different behaviors (different relationships).

### How does each term of equations 1–6 vary with water availability and genetic diversity?

Each of the three approaches of yield provided by Equations (3), (4), and (6) has its own interests, and represents a view of the yield setting. The first equation is more mechanistic and is the one used in all crop models, but the effect of water deficit does not appear explicitly. The second is perhaps more intuitive for understanding the effects of water deficit but can be misleading, depending on the definition that is taken for WUE. The third is the most intuitive, but cannot be related to physiological functions of plants. These three views should be considered as frameworks of analysis to help identify where a given trait is involved in yield formation and what the effects are of a water deficit. The following analyses the contribution of different processes and traits to yield under water deficit. It should be stressed here that none of these traits or function is beneficial or detrimental *per se*. Each trait can be positive, negative, or with negligible effect on the drought scenario (Tardieu, 2012). Hence, tolerance to water deficit and the contribution of traits cannot be considered without considering the drought scenario.

#### Growth reduction of expanding tissues

The first effect of a water shortage is to drastically reduce the growth of expanding tissues, with effects on terms ε_*a*_, *T* and *N*, and indirect effects on other terms. Expansive growth is one of the processes most sensitive to water deficit in leaves, internodes (e.g., the peduncle in cereals) or reproductive organs (e.g., the silks in maize or tubers in potatoes) (Boyer, 1970; Saab and Sharp, 1989; Muller et al., 2011; Tardieu et al., 2011). This occurs because turgor—the driving force for cell expansion—is reduced in the case of water deficit, but also because of other indirect processes such as a reduction in cell division rate or in the extensibility of cell walls (Cosgrove, 2005). A water deficit during the vegetative stages affects leaf growth and hence light interception but, in most species, it also affects the growth of immature storage organs (seeds or tubers).

Via this mechanism of reduced growth, a water deficit can affect the term ε_*a*_ of Equation (3), because it reduces LAI and, therefore, both light interception (Equation 3) and transpiration (Equation 4). The reduction in growth also affects the number of reproductive organs and their abortion ratio (*N* and A, respectively in Equation 6) via a reduction in biomass accumulation (Gambin and Borras, 2007), but also because vegetative and reproductive growth can depend on common mechanisms and common genetic determinists (Welcker et al., 2007). Other terms can also be affected by a reduction in growth, in particular HI if young reproductive organs or young tubers abort. In this case, biomass cannot accumulate in harvested organs in later stages of the crop cycle, and is stored in stems or roots.

There is a very large genetic variability of the sensitivity of growth to water deficit. For instance, quantitative trait loci (QTLs) have been identified for the degree of maintenance of growth under water deficit of leaves (Welcker et al., 2011), silks (Ribaut et al., 1997), or the peduncle (Maccaferri et al., 2008). The sensitivity of leaf growth to evaporative demand and to soil water deficit, which can be determined in controlled conditions, translates into differences in leaf area and into biomass accumulation observed in the field (Chenu et al., 2008). Several QTLs for growth maintenance have been shown to translate into QTLs for yield (Ribaut et al., 1997).

The beneficial effect of growth reduction is to decrease transpiration rate, thereby saving water for the end of the crop cycle. This is favorable under severe terminal water deficit, but is detrimental under mild deficit because of the decrease in cumulated photosynthesis (Tardieu, 2012).

#### Stomatal closure

A second effect of water shortage is to close stomata, thereby affecting the terms ε_*b*_, WUE, *N*, and *W*_*r*_. Plants subjected to water deficit close their stomata, with the involvement of hydraulic and chemical messages such as the plant hormone abscisic acid (ABA) (Tardieu and Simonneau, 1998). This reduces the loss of water by the plant, thereby saving soil water and improving leaf water status, but also reducing the rate of photosynthesis and increasing leaf temperature. These effects can be measured via gas exchange equipment, but measurement of leaf temperature can provide a convenient surrogate for gas exchange if used carefully (Jones, 2007; Guilioni et al., 2008).

There is some genetic variability for stomatal sensitivity to water deficit, but probably less marked than that for growth maintenance. In contrast, there is an interesting genetic variability for WUE, when defined as the ratio of photosynthesis to transpiration rate. Genetic analyses and breeding programmes have been carried out on WUE via its relation to carbon isotope discrimination, resulting in appreciable gains in yield in wheat grown in dry conditions (Condon et al., 2004).

A water deficit decreases the term ε_*b*_ of Equation (3), because the plant accumulates less biomass per unit leaf area than a well-watered plant. This is reversible, because stomata can reopen when more water is available after a rain or watering. In contrast, the heat stress caused by stomatal closure can result in permanent damage to the photosynthetic apparatus, thereby decreasing ε_*b*_ for the rest of the crop cycle. Conversely, WUE usually increases with stomatal closure, because photosynthesis and stomatal conductance are linked with a non-linear relationship. The reduction in photosynthesis affects kernel weight and also the proportion of reproductive organs that develop into seeds. In a number of species, the latter is related to sugar metabolism (Zinselmeier et al., 1999).

The advantages and drawback of an early stomatal closure are similar to those of a reduction in leaf area. This reduces transpiration rate and soil water depletion, but also cumulated photosynthesis. It is therefore advantageous only in case of severe deficit. However, an early stomatal closure has an additional drawback, namely leaf heating. Transpiration contributes to maintain leaves at temperatures compatible with their metabolism, so stomatal closure causes an increase in leaf temperature. One can therefore avoid a water shortage at the cost of a heat stress (Tardieu, 2012).

#### Duration of the crop cycle

A third effect of water deficit is to affect the duration of the crop cycle, thereby affecting the terms Σ, *T*, and *W*. In most species, water deficit affects the duration of the crop cycle by accelerating senescence. This is due to an early expression of genes associated with remobilization of proteins, which are redirected from leaves to reproductive organs (Pic et al., 2002). This reduction in the duration of the crop cycle is an adaptive mechanism, since it allows the plant to complete its cycle earlier while there is still water in the soil, and redirects assimilates to the reproductive organs. This reduces the total intercepted light and, therefore, the biomass accumulation in Equation (3), and the total transpiration in Equation (4). It may also affect the seed weight (Equation 6) if the seed number is not reduced in the same proportion as the reduction in biomass accumulation.

Genetic variability exists in the degree of maintenance of the green leaf area (Borrell et al., 2000), and a breeding strategy has been developed, aimed at maintaining photosynthesis in leaves for a longer duration (“stay-green”). This strategy is adequate for soils with an appreciable water reserve, and may otherwise cause severe stress at the end of the crop cycle through increased transpiration (Hammer et al., 2006).

### Genetic strategies for yield maintenance under water deficit

The above paragraphs provide an understanding of possible strategies for improving yield under water deficit. They also suggest that the maintenance of biomass accumulation under water deficit should be considered as an optimization process between transpiration, biomass accumulation, and its partitioning between root and shoot, rather than as a tolerance process *per se*. It follows that a given trait can have positive, null, or negative consequences, depending on the drought (Chapman et al., 2003; Hammer et al., 2006; Vargas et al., 2006; Tardieu, 2012).

#### Escape strategy

The escape strategy consists of adapting the crop cycle to water availability and evaporative demand, usually by reducing its duration, thereby reducing the total demand for water and avoiding severe terminal stresses. It leads farmers to choose species and genotypes according to local environmental conditions. It is also a strategy adopted by some desert plants that have a very rapid cycle after rain, and finish this cycle before the occurrence of water deficit. For a given genotype, it also consists of reducing the duration of the cycle, thereby reducing the total demand for water and avoiding severe terminal stresses. This strategy saves water but also reduces the accumulated photosynthesis during the crop cycle (Equation 3). Therefore, it consists of a trade-off between a lower risk of terminal stress against a reduced potential yield.

#### Avoidance strategy

The avoidance strategy consists either of the maintenance of transpiration rate under water deficit achieved by improving the size, architecture, or hydraulic conductance of the root system (de Dorlodot et al., 2007) or a reduction in the demand for transpiration by stomatal closure or reduction in leaf area.

***Maintenance of transpiration rate under water deficit via the root system.*** This strategy is observed when the improvement of the root system increases access to soil moisture, i.e., in deep soils. In contrast, when roots grow in a limited volume of soil because of physical barriers (e.g., a hard layer due to compaction) or chemical barriers (e.g., acid soil), improvement of in ability of the root system to rapidly take up water can be detrimental. This is because soil depletion occurs more rapidly, thereby causing severe stress at the end of the season (Tardieu et al., 1992), and because the assimilates invested in roots would be better invested in other organs. Accordingly, while a number of genetic studies of root systems have shown a positive association between yield and root features (Tuberosa et al., 2002), some programs to improve yield under water deficit have resulted in a reduced root biomass (Bolanos et al., 1993; Bruce et al., 2002), or decreased conductivity of the root system (Richards and Passioura, 1989). Therefore, this strategy is a trade-off between a greater carbon investment in roots against an expectation of higher water uptake, which only occurs if soil properties allow the higher uptake.

***Reduction in transpiration by stomatal closure or reduction in leaf area.*** Stomatal closure and reduction of leaf growth rate under water deficit has been selected by evolution to reduce the risk of failure at the end of the growing season, because they both reduce the plant demand for water. However, they intrinsically reduce the yield expectation by decreasing the proportion of light intercepted by leaves (ε_*a*_, Equation 3), and/or the efficiency of the transformation of light into biomass, which follows stomatal conductance (ε_*b*_, Equation 3). It is noteworthy that many experiments in pots that identify “drought-tolerant” plants, in fact, use this strategy (e.g., Iuchi et al., 2001) Therefore, this strategy trades off a lower risk of plant failure against lower potential biomass production.

#### Growth maintenance

Unlike in the other strategies described so far, that of growth maintenance consists of continued growth of the most important organs, thereby maintaining yield. However, the maintained transpiration may cause a crop failure at the end of the crop season. Therefore, this strategy exchanges the maintenance of yield potential for a high risk of crop failure.

***Maintenance of leaf growth.*** The maintenance of leaf growth under water deficit allows better light interception, thereby increasing photosynthesis but also increasing the transpiration rate and soil water depletion. Therefore, it is appropriate in many cases, although not for severe, terminal water deficits. It is noteworthy that, in one mapping population, half of the QTLs for sensitivity of leaf growth overlapped with those of silk growth (Welcker et al., 2007), suggesting that mechanisms favoring expansive growth may also favor reproductive development.

***Maintenance of reproductive growth.*** The maintenance of reproductive growth around the time of flowering allows the maintenance of capacity for storage of photoassimilates later in the crop cycle, thereby increasing HI (Equations 3, 4) and decreasing A, the proportion of aborted reproductive organs (Equation 6). This strategy has been successful in several species, in particular maize, via the assessment of the anthesis-silking interval (ASI), which is typically increased by water deficit and negatively correlated with yield. Phenotypic selection under well-managed stress environments for low ASI has produced large genetic gains and resulted in significant impacts (Bolanos and Edmeades, 1993b; Ribaut et al., 2004).

#### Increase in water-use efficiency

An increase in WUE may seem to be the ideal candidate mechanism for drought-prone environments. In crops, WUE has been regarded as a “conservative strategy” involving reduced transpiration, such that the positive influence of a higher WUE on yield may be reduced under moderately favorable environments and become a penalty under the most favorable conditions (Rebetzke et al., 2002; Richards et al., 2002). This strategy has been used in wheat for Australian environments, where water must be used conservatively to allow the crop to complete its life cycle. It has led to the release of two cultivars (Condon et al., 2004).

#### Increase in harvest index

Finally, an increase in HI (Equations 3, 4) has been a major way of increasing yield, even under water deficit (Turner, 1997). Furthermore, a change in biomass allocation between stem, roots, and seeds has been a clear route to progress.

## The progression of developmental stages of a plant can be predicted by using thermal time

### Why use thermal time and plant development models?

The above paragraphs show that environmental stress has different consequences depending on the phenological stage at which it occurs in the plant. In particular, some stages such as flowering present a higher sensitivity to stresses, while others such as grain filling present a lower sensitivity. It follows that a genetic comparison can be biased if the stress occurs at different stages in each genotype, because some genotypes will encounter the stress at a sensitive stage while others will encounter it at a stage with lower sensitivity. This results in non-reproducible experiments.

Therefore, it is essential that the main phenological stages of each genotype are precisely recorded. This raises two problems. The first is that, because a key stage such as flowering can occur over one or more weeks in a population of genotypes, it is usually impossible to visit each day to obtain the flowering date of every individual genotype. The second is that some key stages are difficult and lengthy to determine. While emergence, leaf number or flowering time can be obtained in a straightforward way, determining other stages such as flower initiation requires a detailed analysis. However, these stages can often be determined from other phenological stages such as the number of leaves.

When an experiment is repeated in naturally fluctuating conditions, phenological stages occur at different dates in each experiment. The number of days after emergence cannot, therefore, provide a good prediction of the stages. For instance, the progression of leaf initiation on the stem generally differs between different experiments in the field or in the greenhouse (Granier and Tardieu, 1998; Granier et al., 2002). Therefore, we need a tool that can: (1) simulate the exact date of a given stage from several datapoints obtained at different dates; (2) compare the behavior of a given genotype in different experiments; and (3) estimate the dates of “hidden” stages, e.g., flower initiation or the beginning of stem elongation, from other stages that are easier to determine.

### What is thermal time?

#### Rates are related to organ temperature with stable relationships

**Figure 4:**
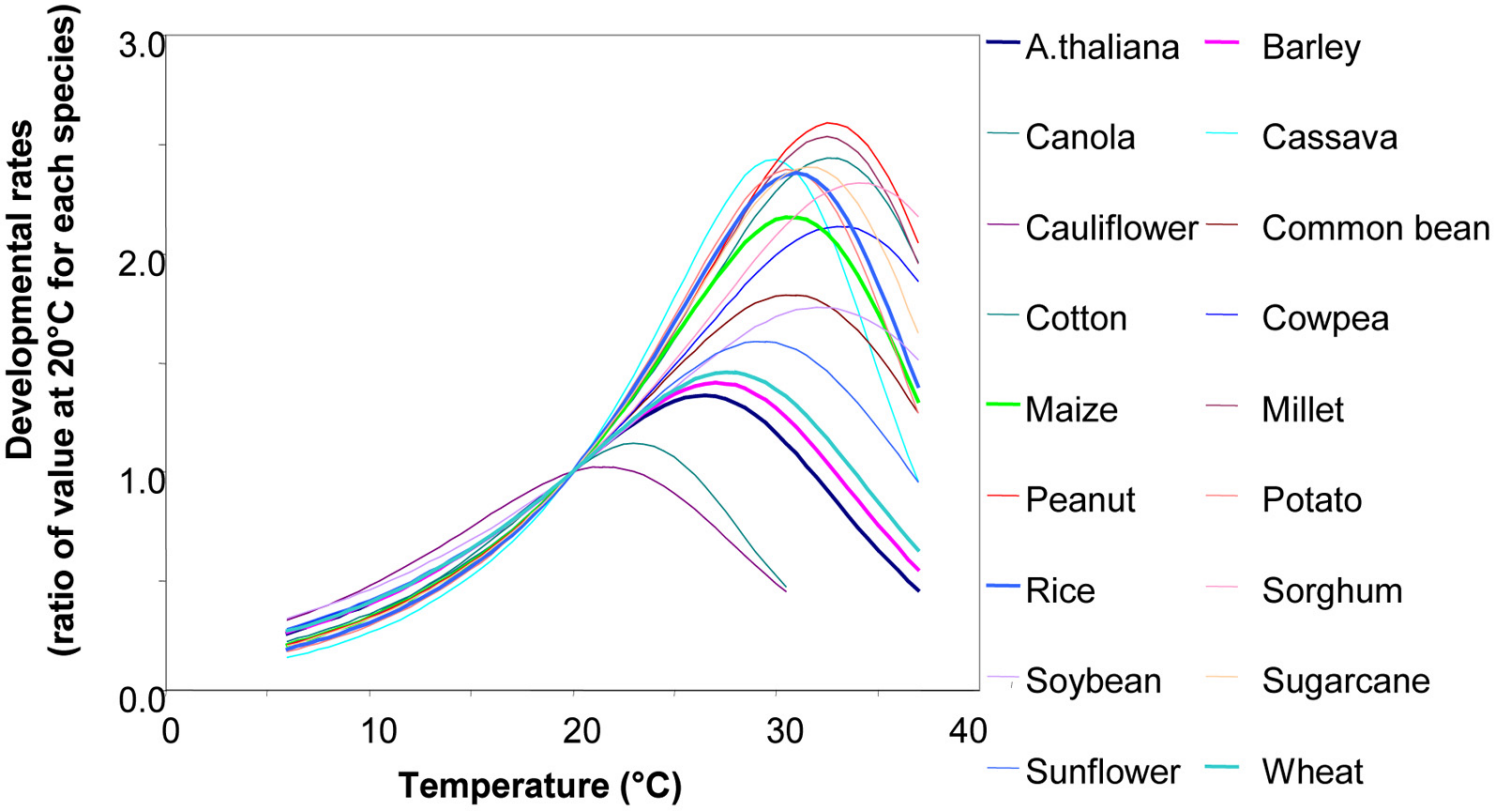
**The relationship between temperature and developmental rates for 18 species, e.g., leaf appearance rate, leaf or root elongation rates, germination rates, and reciprocal of the duration of cycle phase (but *not* photosynthetic rate and enzymatic rates involved in carbon metabolism).** Rates are presented as a ratio of their value at 20°C, so thermal time can be expressed as equivalent days at 20C°. Redrawn from Parent and Tardieu (2012). Data and equations can be found in Parent and Tardieu (2012) (SI). See also Parent et al. (2010a,b).

Biological processes have a rate that follows temperature, with a non-linear relation that resembles the enzymatic responses to temperature (Figure 4) (Parent et al., 2010a; Parent and Tardieu, 2012). However, in a restricted range of temperature, this relationship can be considered as linear in pea (Turc and Lecoeur, 1997) and sunflower (Granier and Tardieu, 1998). In the latter, the same relationship held for plants grown in the growth chamber, in the greenhouse or in the field. In a study of the relationship between meristem temperature and maize leaf elongation rate over 15 field experiments, three growth chamber experiments and three greenhouse experiments at night (i.e., in the absence of evaporative demand), using a single genotype (hybrid Dea), the same relationship was found to apply to all three conditions (Tardieu, 2003). Marked differences in slopes between inbred lines were observed consistently over successive experiments. The slope is therefore a stable characteristic of a genotype (Reymond et al., 2003). These relationships only apply during the night. Elongation rates at a given temperature are lower during the day owing to the effect of evaporative demand, which is taken into account by a second relationship.

Several conclusions can be drawn:

- If the same relationship holds for experiments in different places, years, and experimental conditions, this means that the temperature dependence of rates is a stable characteristic of a genotype. One can therefore calculate a common thermal time for all genotypes of a mapping population.
- The relationships presented in Figure 4 imply that rates can be deduced from the temperature. For example, in Figure 4, twice as many leaves will have been initiated at 26°C than at 16°C. Alternatively, it can be stated that the time sensed by the plant elapses twice as rapidly at 26°C as at 16°C. This is the intuitive basis for thermal time: thus, plants “sense” thermal time rather than calendar time, and thermal time depends on temperature. The x-intercept of this relationship is termed the “threshold temperature.” If the relationship were linear across the whole range, this threshold temperature would be the temperature at which the rate is zero. This is not usually the case, since the response tends to be curvilinear at low temperatures, hence the threshold temperature has rather a statistical definition.
- Several processes such as leaf appearance rate, cell division rate, or leaf growth rate have a common relationship with temperature over the whole range 6–35°C (Parent and Tardieu, 2012). This means that thermal time as sensed by several processes or organs is the same. It is, therefore, the “physiological age” of the plant.

#### Calculation of thermal time

Thermal time depends on the existence of the linear relationships described above. The first relates temperature to the rates of processes involved in leaf growth:

(7) $${\text{dP/dt = a (T - T}}_{\text{0}}\text{)}$$

where *P* is the studied process (e.g., expansion, cell division, or leaf initiation), *T* is the current temperature, *a* and *T*_0_ are the slope, and the x-intercept, respectively, of the relationship between *dP/dt* and *T*. The second relationship involves the reciprocal of the duration of the studied process:

(8) $${\text{1/d = b (T - T}}_{\text{0}}\text{)}$$

where *d* is the time during which expansion (or any other developmental process) occurs in a given leaf, or the time during which leaf initiation occurs on the apex. It follows that, at time *d*:

(9) $$P=a\sum_{0}^{d}\left(T-T_{0}\right)dt$$

∑^*d*^_0_(*T* − *T*_0_) *dt* is commonly named thermal time (unit of °Cd, when calculated with a daily timestep).

This calculation can easily be carried out using a spreadsheet, where each line represents a date. First, the mean temperature for each day must be calculated. An efficient way for that is to consider the average of the maximum and minimum temperatures, which are usually available in weather stations. The thermal time elapsed during a given day is the difference between the mean temperature of the day and threshold temperature of the considered species. This is available in the literature (e.g., 10°C for maize, 11°C for sorghum, 13°C for rice, etc.). The thermal time for a given period of time is the sum of the thermal times of all days in question.

However, when the temperature sensed by plants decreases below 15°C or reaches temperatures higher than the optimum temperature (see optimum temperatures of main crop species in Parent and Tardieu, 2012), the calculation presented above can cause serious bias. In this case, and in the general case for some species-like rice, another calculation of thermal time should be preferred, which takes into account the plant response in the whole range of temperature. This alternative method is presented in Parent and Tardieu (2012) and Parent et al. (2010b), with associated spreadsheets. Crop models such as APSIM use a series of linear relations that approximate the general relation (Hammer et al., 2010).

### The development of plants follows a programme that is stable for a given genotype

**Figure 5:**
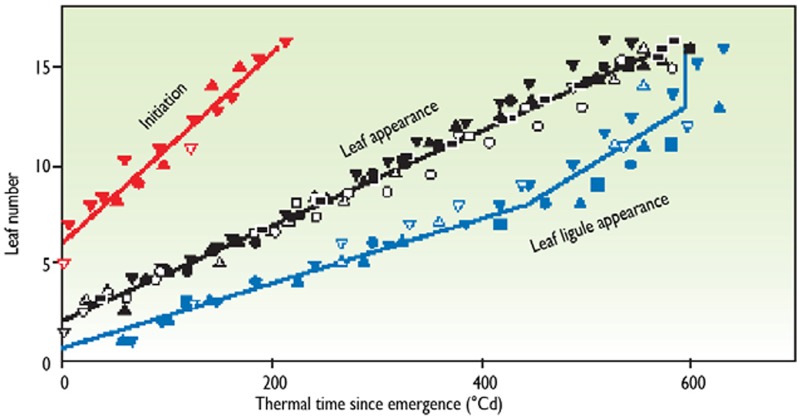
**Example of a model of plant development in maize.** Initiation, leaf appearance, and ligule appearance in maize are expressed as a function of thermal time. The y axis represents each position of the leaf on the stem. For each leaf, the beginning and end of linear elongation occurred at a common thermal time in all experiments (redrawn from Chenu et al., 2008).

A model of plant development can be built at the whole plant level, using the method presented previously. The occurrence of several phenological stages of the plant can be predicted, depending on thermal time. For example, Figure 5 (Chenu et al., 2008) presents the number of leaves that have been initiated, the number that are visible, and the number that have stopped growth as a function of thermal time after emergence. Presented relations summarize different experiments and different plants in each experiment. For instance, leaf 10 was initiated at 90°Cd, was visible at 320°Cd, and ceased elongation at 490°Cd. If we consider all experimental points, three regression lines appear which allow prediction of the phenological stages. It can therefore be assumed that, in any experiment in any place in the world, leaf 10 of this genotype stops elongation 490°Cd after emergence. As an example, it has been checked that a common development model for sorghum was valid in both Mali and in Montpellier, France (Lafarge and Tardieu, 2002).

The model of plant development summarized in Figure 5 can be read in two ways. First, if considered vertically, for instance at 400°Cd, it indicates the number of initiated leaves and leaves that have ceased expansion on that day. Thus, it is a “snapshot” of the plant on a given day. Then, if considered horizontally, it indicates the development of each organ. If a phenological stage has not been recorded exactly when it occurred, it can be inferred from measurements carried out before and after the date in question. For instance, if the date of plant emergence occurs between two visits to the experiment, it can be reconstructed by recording the leaf number at 2 or 3 dates, and calculating the date at which the leaf number would be zero.

Several experiments can be analyzed jointly, and the timing of stresses of each experiment, and each genotype can be placed on a single scale of development. This is of considerable help in the interpretation of a network of experiments, which would otherwise have apparently erratic behavior.

## A minimum dataset for using methods described here

The above methods allow comparison between experiments, genotypes, and treatments, provided that a minimum set of measurements is collected. This dataset is currently used in several consortium, e.g., http://www.drops-project.eu. These are described below.

### Key dates of the crop cycle

Although this information is relatively straightforward, it is frequently missing, meaning that none of the methods presented above can be used. The most important dates are sowing, emergence, flowering, and physiological maturity (harvest). It is useful to record intermediate stages such as leaf number, which allow recalculation of missing stages by using thermal-time-based interpolation as described above.

### Daily irradiance or photosynthetic photon flux density

This information about available light is essential because: (1) it is an input for calculating the soil water balance; and (2) it allows estimation of the potential biomass accumulation in the environment in question. Irradiance (*I*_*r*_, measured in W m^−2^) is better suited for the first use and is provided by pyranometers, while PPFD (mol m^−2^ s^−1^) is better suited to the second use, and is provided by PAR sensors. Because either variable can be translated into to the other under field conditions, both are acceptable.

Light intensity has a relatively low site specificity. It is acceptable to record data from a weather station located at several kilometer distance provided that: (1) the weather station is in the same geographical situation as the experimental field (altitude etc.); and (2) there can be reasonable confidence in the data (especially if missing data are not too frequent, if the sensors are of satisfactory quality, etc.). In contrast, special care has to be taken in greenhouse and growth chamber experiments because of the high spatial variability (both horizontal and vertical) of light in these environments. A map of light intensity, or at least the use of several sensors, is recommended.

### Air temperature

Together with irradiance, information on the air temperature (*T*) is necessary for calculating the soil water balance. It also allows estimation of thermal time if plant temperature is close to air temperature. This is usually acceptable for well-watered adult plants, but is prone to large errors during early phases in monocot species and in plants subjected to water deficit. It allows estimation of the occurrence of high temperature stresses (e.g., at *T* > 40°C), of risks of oxidative stresses (e.g., at *T* < 3°C and PPFD > 1000 mol m^−2^ s^−1^), and of phenological stages, with the use of thermal time. This information must be recorded close to the experimental field using a local weather station or a data logger with thermocouples. Data can be recorded at daily intervals as minimum and maximum temperatures. The data need to be measured at plant height in greenhouse or growth chamber experiments.

### Air relative humidity, vapor pressure deficit, and reference evapotranspiration

These three variables quantify the evaporative demand, which is essential for estimating stress levels, for characterization and for calculation of the soil water balance. They provide essentially the same information, but with different time scales and usefulness. Relative humidity (RH), expressed as a percentage and VPD (in kPa) are calculated on short timescales (minute to hour), and ET_0_ (in mm per day) is on a daily timescale. The variable recorded in the database would be ET_0_, either calculated from other climatic data (*I*_*r*_, VPD, and *T* wind speed) recorded in a datalogger (see above), or directly calculated by the weather station. ET_0_ is species-independent and calculated by energy balance.

RH and wind speed have relatively low site specificities. As in the case of air temperature, it is acceptable to record these data from a weather station located at several kilometer distance. RH in greenhouse and growth chamber experiments should be recorded with replications, because of the large spatial variability. A method for calculating ET_0_ is available at: http://www.fao.org/docrep/X0490E/x0490e04.htm#reference%20crop%20evapotranspiration%20(eto).

It might be useful here to emphasis on two frequent errors:

- RH should not be interpreted *per se*, because it does not characterize the evaporative demand when the air temperature is fluctuating. The use of both RH and air temperature allow a very simple calculation of VPD, which is the driving force for transpiration. Extreme events such as the sirocco should be recorded as daily maximum VPD over a period of 3 or 4 h.
- Mean daily air VPD or RH recordings are not acceptable for characterizing the daily evaporative demand; ET_0_ should be used.

### Rainfall and irrigation

Recordings should be made near the field (<300 m distant) because of very high spatial variability. Simple rain gauges are efficient and inexpensive but require frequent visits, while automatic rain gauges connected to a datalogger are more expensive but are useful in distantly located experiments.

### Initial soil water content in the field

The water balance data begin at a given date (e.g., emergence), at which time the soil water content must be recorded. This can be done with augers over a depth similar to the final rooting depth, with particular attention to spatial variability in the field. This measurement is important especially in experiments where the rainfall is zero or negligible. Some “shortcuts” can be acceptable, especially when either the rainfall or irrigation before the experiment is sufficient to guarantee that the soil is at retention (or field) capacity.

### Soil hydraulic properties

These are essentially the variables that allow calculation of the limits of soil water reserves, namely the field capacity and the limit of water extraction. These should be measured in experimental fields where drought experiments often take place, using equipment that measures soil water content, e.g., neutron probes or time domain reflectometry (TDR). These properties can also be inferred from the soil texture (e.g., loamy sand, clay loam, etc.) and the estimated rooting depth.

### Light interception

With current techniques, it is usually not feasible to measure LAI of all genotypes in an experiment. LAI can be measured by collecting all leaves on a sample soil area and measuring their area. It can also be measured indirectly and non-destructively using sensors that directly measure the proportion of intercepted light. Finally, novel imaging technique with NDVI remote sensing will shortly allow one to measure leaf area of all genotypes in an experiment.

## Concluding remarks

It is not possible to present in detail here how to use each method for each species However, it can be stressed that the tools presented here help in the interpretation of data gathered from networks of experiments, and that they are the base of all existing plant models.

The potential production of each site can be calculated from the development model, which provides an estimate of leaf area, and the available light. For instance, the biomass accumulation in cloudy years is lower than that of bright years, if water is not seriously limiting yield. In the same way, a hot year reduces yield even in the absence of heat stress or water stress, by reducing the duration of the crop cycle. It is particularly useful to compare the potential productivity of experimental sites and years, in order to distinguish the natural variability of yield linked to light availability from the effects of stressing events.

The soil water balance can be calculated for each genotype, provided that a minimum dataset has been collected. This requires estimation of the change with time in leaf area of each genotype. The latter information can be inferred from measurements of “probe genotypes” having approximately the same cycle duration as a class of genotypes under examination. Once leaf area has been estimated, it is possible to calculate the proportion of evapotranspiration needed by the genotype in question in comparison with the reference level of evapotranspiration.

### Conflict of interest statement

The author declares that the research was conducted in the absence of any commercial or financial relationships that could be construed as a potential conflict of interest.
